# An O-Specific Polysaccharide *Shigella flexneri* 3a Conjugate Vaccine is Immunogenic and Protective against Virulent Keratoconjunctival Challenge in Guinea Pigs

**DOI:** 10.4269/ajtmh.25-0269

**Published:** 2025-08-05

**Authors:** Jeshina Janardhanan, Chanchal Wagh, Jibing Yang, Richelle C. Charles, Ruchir Kumar Pansuriya, Fahima Chowdhury, Robert W. Kaminski, Ashraful Islam Khan, Taufiqur Rahman Bhuiyan, Firdausi Qadri, Pavol Kováč, Peng Xu, Edward T. Ryan

**Affiliations:** ^1^ Division of Infectious Diseases, Massachusetts General Hospital, Boston, Massachusetts;; ^2^ Center for Comparative Medicine, Massachusetts General Hospital, Boston, Massachusetts;; ^3^ Department of Medicine, Harvard Medical School, Boston, Massachusetts;; ^4^ Department of Immunology and Infectious Diseases, Harvard T.H. Chan School of Public Health, Boston, Massachusetts;; ^5^ International Vaccine Institute, Seoul, South Korea;; ^6^ International Centre for Diarrhoeal Disease Research, Bangladesh, Dhaka, Bangladesh;; ^7^ Latham BioPharm Group, Cambridge, Massachusetts;; ^8^ NIDDK, LBC, National Institutes of Health, Bethesda, Maryland

## Abstract

*Shigella* infection is a major cause of diarrhea, cognitive and physical stunting, and death in young children in resource-limited settings. A vaccine that is protective against shigellosis is needed. Immune responses that target the O-specific polysaccharide (OSP) of *Shigella* spp. are protective against shigellosis. We previously reported the development and evaluation of a conjugate vaccine targeting *Shigella flexneri* (*S. flexneri*) 3a in mice. Here, we report the evaluation of this vaccine (*Shigella* conjugate vaccine *S. flexneri* 3a OSP conjugated to a 52 kiloDalton recombinant fragment of the tetanus toxin heavy chain [SCV-Sf3a OSP:rTTHc]) in a second animal model: the guinea pig. This vaccine induced prominent OSP-, lipopolysaccharide-, and rTTHc-specific Immunoglobulin (Ig)G, IgA, and IgM responses in the sera of vaccinated animals. *Shigella* conjugate vaccine *S. flexneri* 3a also induced serum bactericidal functional antibody responses, and vaccinated guinea pigs were protected against a virulent strain of keratoconjunctival challenge in the standard *Shigella* Sereny assay. These results support the further development of SCV-Sf3a OSP:rTTHc.

## INTRODUCTION

*Shigella* infection is a major cause of diarrhea-related illness and death, especially in children under 5 years of age in low- and middle-income countries.^1^^,^^2^ *Shigella* spp. can be categorized by species and serotype, largely based on the structure of the O-antigen, O-specific polysaccharide (OSP). Based on global distribution and antigenic structure, it has been proposed that a vaccine effective against *Shigella flexneri* (*S. flexneri*) 2a, *S. flexneri* 3a (Sf3a), *S. flexneri* 6, and *Shigella sonnei* (*S. sonnei*) could protect against ∼85% of global shigellosis.^3^^–^^5^ Protection against shigellosis is largely species- and serotype-specific, and these responses target the OSP component of bacterial lipopolysaccharide (LPS).^3^^–^^5^ We have previously described the development of a *Shigella* conjugate vaccine, including the OSP of Sf3a (SCV-Sf3a OSP:rTTHc).^6^ This vaccine contains the OSP of Sf3a conjugated to a recombinant fragment of the tetanus toxin heavy chain (rTTHc).^6^ This vaccine induced OSP-, LPS-, and rTTHc-specific IgG responses, as well as OSP- and LPS-specific IgM responses, in vaccinated mice.^6^ The vaccine also induced functional bactericidal antibody responses in mice and provided protection against lethal intraperitoneal challenge with virulent Sf3a.^6^ Here we report the evaluation of SCV-Sf3a OSP:rTTHc in guinea pigs. The guinea pig keratoconjunctivitis (Sereny) model was one of the first established animal models for studying the virulence of *Shigella* strains and remains a widely used model for investigating vaccine-related protection from *Shigella* spp. infection.^7^^–^^9^

## MATERIALS AND METHODS

### Bacterial strains and reagents.

The SCV-Sf3a OSP:rTTHc vaccine used in this study contained four Sf3a OSPs conjugated to each rTTHc moiety, with an average repeating unit length of 12–13 for each Sf3a OSP.^6^ Virulent Sf3a strain J17B was used in the Sereny assay, and Sf3a Sf3a050214_5 was used in bactericidal assays.^6^ Both strains were grown in tryptic soy broth (Becton Dickinson, Sparks Glencoe, MD) and on tryptic soy agar plates (Becton Dickinson).

### Vaccination and collection of samples.

Female Hartley guinea pigs, ranging in weight from 200 to 250 g, were purchased from Charles River Laboratories International, Inc. (Wilmington, MA) and allowed to acclimate for 7 days after arrival at the institutional facility. To assess the immunogenicity of SCV-Sf3a OSP:rTTHc, the guinea pigs were divided into two groups of five animals each. A control group received phosphate-buffered saline (PBS) mixed at a 1:1 (v/v) ratio with alum (Adju-Phos® adjuvant, InvivoGen, San Diego, CA); the test group received SCV-Sf3a OSP:rTTHc (containing 20 *µ*g of the polysaccharide component) mixed with alum at a 1:1 (v/v) ratio. The animals were inoculated intramuscularly in the quadriceps of the right thigh. To assess the kinetics of immune responses, the animals received intramuscular injections of alum or vaccine and alum on days 0, 21, and 42, followed by a booster dose on day 67. Blood was collected from the lateral saphenous vein on days 0, 7, 21, 28, 49, 56, and 76, and sera were separated via centrifugation and stored at –80°C until analysis.

### Antigen-specific antibody responses in sera.

Sera samples were used to assess OSP-, LPS-, and rTTHc-specific Immunoglobulin (Ig)G, IgM and IgA responses using standard ELISA protocols, as previously described.^6^^,^^10^^–^^12^ Briefly, ELISA plates were coated with Sf3a OSP: bovine serum albumin (OSP:BSA; 100 ng/well), LPS (250 ng/well), or rTTHc (100 ng/well) in 50 mM carbonate buffer.^6^ Sera samples were diluted to a ratio of 1:250, in 0.1% PBS Tween 20 (PBS-T; Sigma-Aldrich, St. Louis, MO) and added at 50 *µ*L per well. The presence of anti-OSP, anti-LPS, or anti-rTTHc antibodies was detected by using horseradish peroxidase-conjugated goat anti-guinea pig IgG (Southern Biotech, Birmingham, AL), rabbit anti-guinea pig IgM (BIORBYT LLC, Durham, NC), or sheep anti-guinea pig IgA antibody (Invitrogen, Waltham, MA) diluted 1:1000 in 0.1% BSA in PBS-T. Plates were developed and read as described previously.^6^ A responder was defined as having a twofold increase in antigen-specific antibody responses compared with day 0 and a higher optical density value observed at any time point in control animals.

### Serum bactericidal responses.

Serum bactericidal antibody titers against the Sf3a050214_5 strain were assessed using a micro-assay, as previously described.^6^ The bactericidal titer was calculated as the reciprocal of the lowest dilution of serum associated with a 25% reduction in optical density compared with wells containing no serum; a responder was defined as having a ≥4-fold increase in bactericidal reciprocal end-dilution titer compared with the baseline.

### Guinea pig challenge model.

Two weeks after receiving the booster vaccination, the animals underwent the Sereny keratoconjunctival assay to assess protection against virulent challenge at a mucosal–epithelial surface.^13^^–^^15^ Briefly, guinea pigs were first anesthetized by using a mixture of 100 mg/kg ketamine (Dechra Veterinary Products, Leawood, KS) and 10 mg/kg xylazine (Dechra Veterinary Products), administered intramuscularly. Then, 20 *µ*L of a bacterial suspension containing ∼10^9^ colony forming units of virulent Sf3a J17B was administered into the lower conjunctival sac of one eye with a soft micropipette. Normal saline was administered into the other eye as a control. Animals were observed daily for 1 week to monitor the development of keratoconjunctivitis, characterized by conjunctival edema, inflammation of the conjunctiva, and purulence in the eyelid.^16^^,^^17^ The severity of keratoconjunctivitis was scored as follows: 0 = no reaction or only mild irritation; 1 = mild conjunctival injection with minimal discharge; 2 = nonpurulent keratoconjunctivitis with moderate discharge (did not recur within 5 minutes of wiping away); 3 = purulent keratoconjunctivitis involving significant conjunctival infection with purulent discharge (recurred within 5 minutes of wiping away discharge), extensive edema, and periorbital swelling.

### Statistics and graphs.

Statistical analyses were performed using GraphPad Prism 10 (GraphPad Software, Inc., Boston, MA). Data from different groups were compared using Mann–Whitney *U* tests. All reported *P*-values were two-tailed, with a cutoff of *P* <0.05 used as a threshold for statistical significance.

## RESULTS

### Immunogenicity of SCV-Sf3a, including bactericidal activity and protection in the Sereny assay.

**Figure 1.**
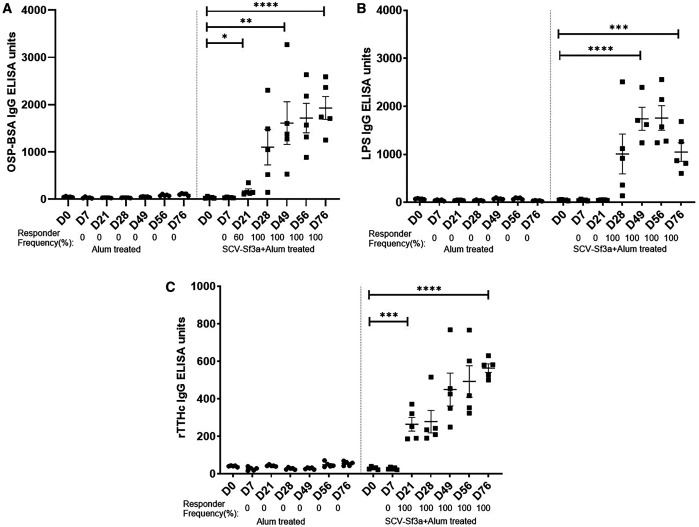
Serum IgG responses against (**A**) *Shigella flexneri* 3a (Sf3a) O-specific polysaccharide (OSP), (**B**) Sf3a lipopolysaccharide, and (**C**) the recombinant fragment of the tetanus toxin heavy chain at different time points in two groups of guinea pigs vaccinated with either *Shigella* conjugate vaccine Sf3a with alum or alum alone. Dots represent responses in individual animals. The mean and standard error of the mean are reported for each group. * *P* <0.05; ** *P* ≤0.01; *** *P* ≤0.001; **** *P* <0.0001 (1:250 dilution). Responder frequencies are calculated as >4 times for anti-OSP IgG and >2 times for all others on the basis of the day 0 value.

**Figure 2.**
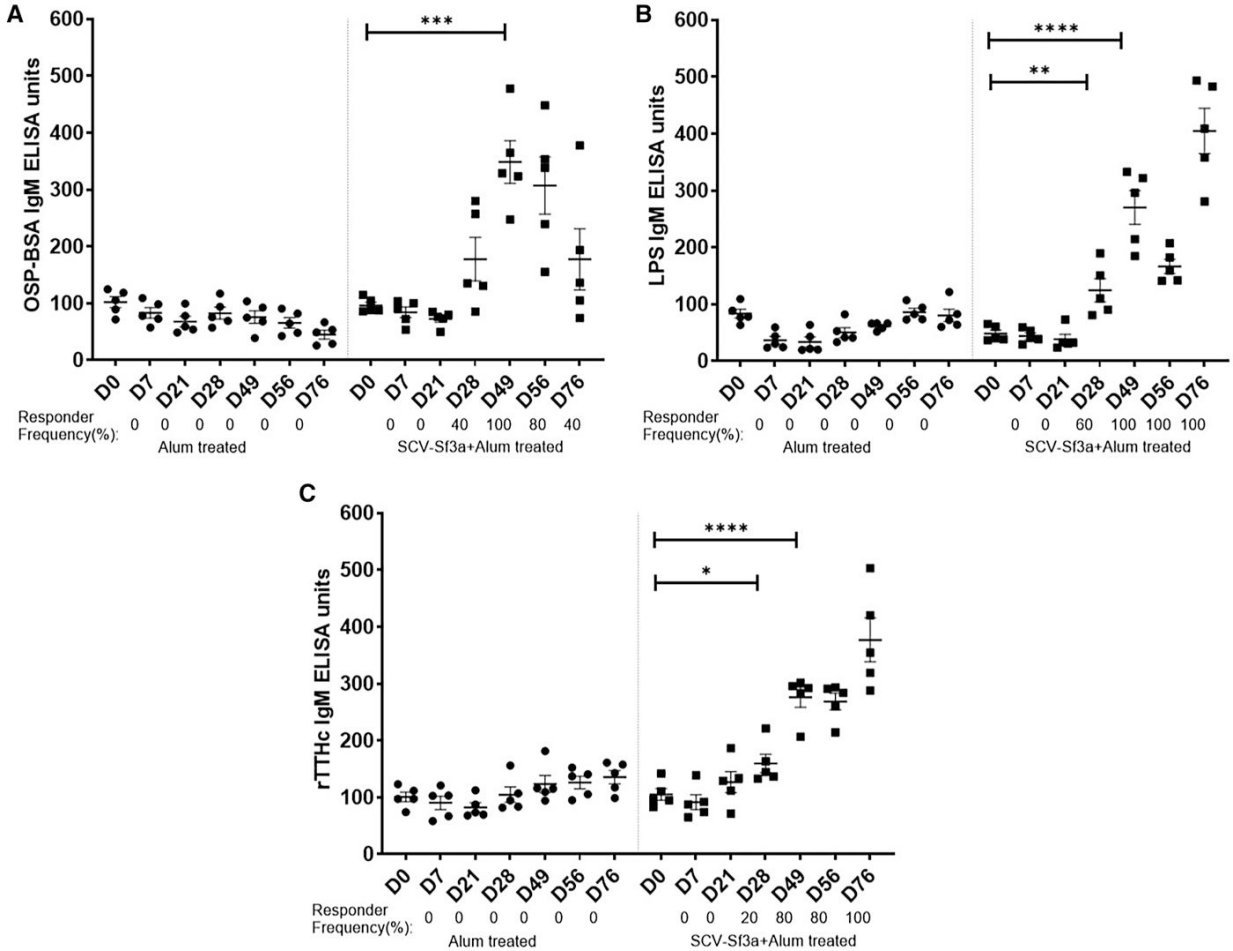
Serum IgM responses against (**A**) *Shigella flexneri* 3a (Sf3a) O-specific polysaccharide, (**B**) Sf3a lipopolysaccharide, and (**C**) the recombinant fragment of the tetanus toxin heavy chain at different time points in two groups of guinea pigs vaccinated with either *Shigella* conjugate vaccine Sf3a with alum or alum alone. Dots represent responses in individual animals. The mean and standard error of the mean are reported for each group. * *P* <0.05; ** *P* ≤0.01; *** *P* ≤0.001; **** *P* <0.0001. Responder frequencies are also listed (1:250 dilution) Responder frequencies are listed as >2-fold day 0 values.

**Figure 3.**
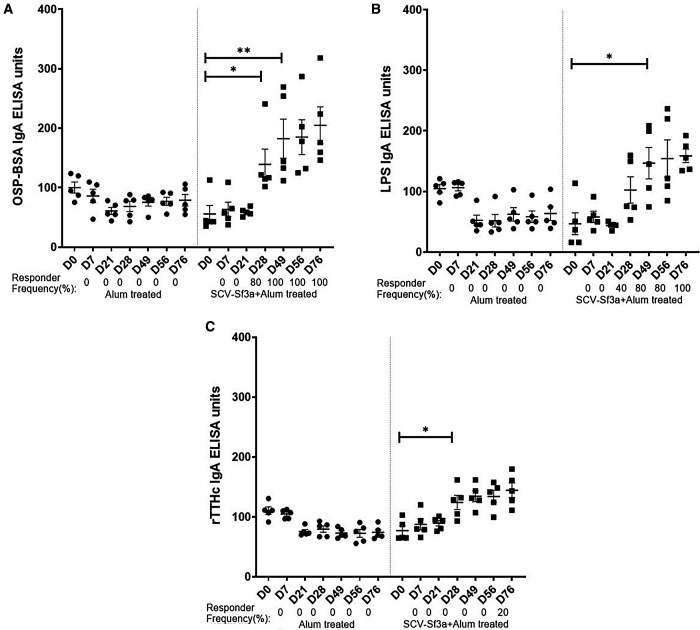
Serum immunoglobulin A responses against (**A**) *Shigella flexneri* 3a (Sf3a) O-specific polysaccharide, (**B**) Sf3a lipopolysaccharide, and (**C**) the recombinant fragment of the tetanus toxin heavy chain at different time points in two groups of guinea pigs vaccinated with either *Shigella* conjugate vaccine Sf3a with alum or alum alone. Dots represent responses in individual animals. The mean and standard error of the mean are reported for each group. * *P* <0.05; ** *P* ≤0.01 (1:250 dilution). Responder frequencies are listed as >2-fold day 0 values.

**Figure 4.**
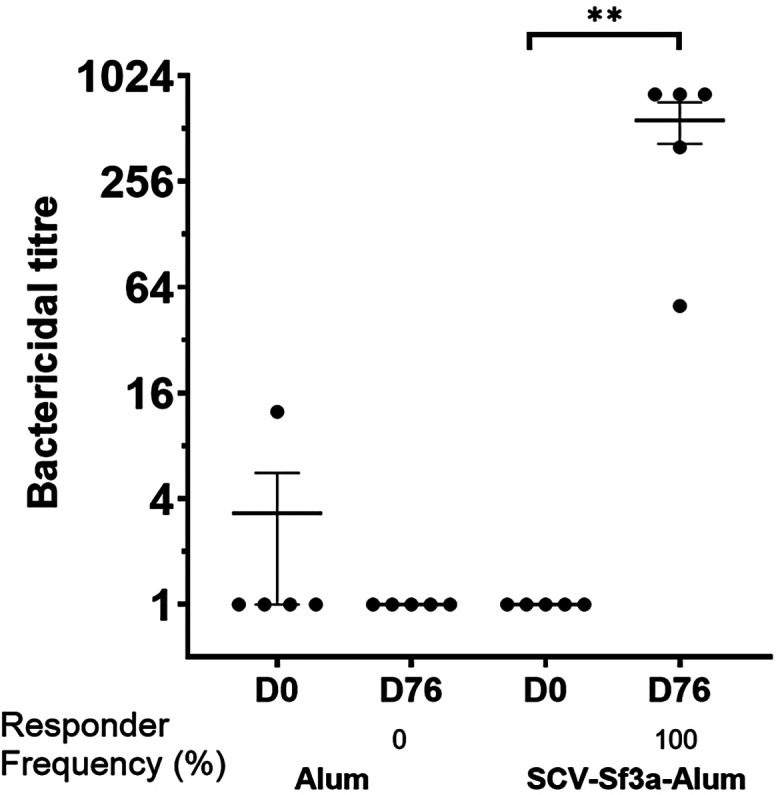
Bactericidal responses in vaccinated cohorts of guinea pigs. Dots represent responses in individual animals on days 0 and 76. The mean and standard error of the mean are reported for each group. ** *P* ≤0.01 from baseline (day 0) response. Responder frequencies are listed for ≥4-fold increases in bactericidal reciprocal end-dilution titer compared with the baseline.

**Figure 5.**
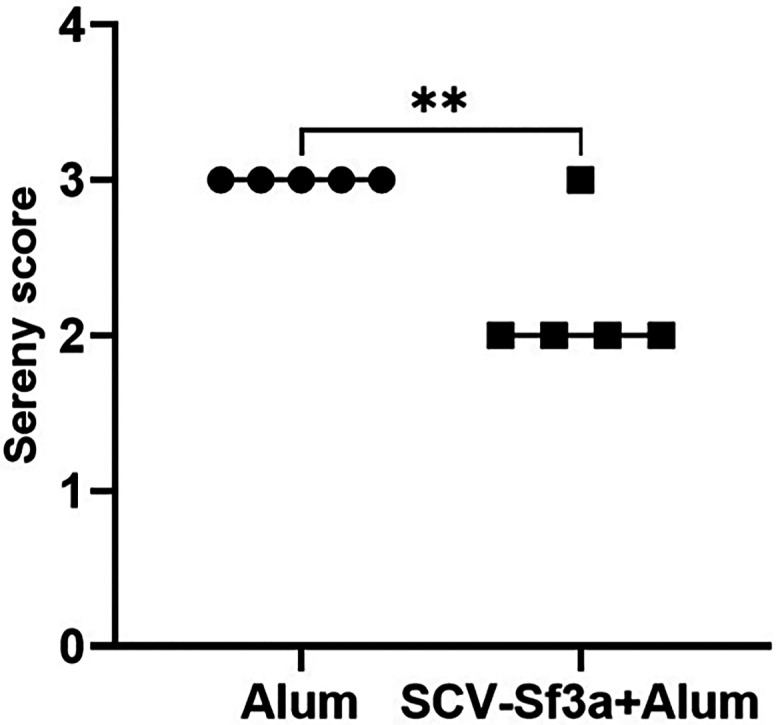
Assessment of protection afforded by vaccination with *Shigella* conjugate vaccine with *Shigella flexneri* 3a (SCV-Sf3a) using a Sereny assay. Guinea pigs (*n* = 5 per group) were infected with 8 × 10^8^ colony forming units of *Shigella flexneri* 3a virulent strain J17B in the right eye and observed for 72 hours. Animals vaccinated with the SCV-Sf3a vaccine exhibited significantly better Sereny scores (** *P* ≤0.01) compared with the control group.

Guinea pigs vaccinated with SCV-Sf3a OSP:rTTHc and alum developed OSP-specific IgG responses (Figure 1A) after a single dose (responder frequency of 60%). The responder frequency increased to 100% by day 28. Lipopolysaccharide-specific IgG responses were detectable after two vaccinations (Figure 1B), whereas rTTHc-specific IgG responses were evident after only one dose of the vaccine (Figure 1C). The magnitude of the responses largely plateaued after the second dose of the vaccine across all antigens assessed. The SCV-Sf3a OSP:rTTHc and alum treatment also induced OSP-, LPS-, and rTTHc-specific IgM (Figure 2A–C) and IgA (Figure 3A–C) responses in the sera of vaccinated animals. The booster vaccination on day 67 did not further increase responses significantly. Vaccinated animals developed prominent bactericidal responses capable of killing Sf3a isolates (Figure 4) and exhibited lower Sereny scores on the keratoconjunctival challenge assay (Figure 5; *P* <0.01; Supplemental Figure 1), indicating protection against severe disease.

## DISCUSSION

Given the global incidence and increases in antimicrobial resistance, there is an urgent need for vaccines effective against shigellosis. Such vaccines must be protective in young children in resource-limited areas because these children bear the largest global burden of shigellosis. *Shigella* infection is the second leading cause of moderate to severe diarrhea in children 1–2 years of age, as well as the leading cause of diarrheal-related death in children 3–5 years of age globally.^2^^,^^18^^–^^22^ *Shigella* are enteroinvasive bacteria that cause acute inflammatory colitis, and several vaccines targeting shigellosis are being developed.^23^^–^^27^ Protection against *Shigella* infection has been associated with several *Shigella* antigens; however, protective immunity is generally species- and serotype-specific, despite *Shigella* sharing many protein and virulence antigens. Species and serotype specificity are largely defined by the OSP component of the bacterial LPS, and the majority of *Shigella* vaccines in development are designed to induce prominent and durable immune responses against OSP and LPS, with or without attempting to induce immune responses to other antigens.^28^^–^^30^

Because young children do not often mount prominent and durable immune responses against T cell-independent polysaccharide antigens, such as OSP, we are focusing our efforts on developing OSP-conjugate vaccines. A similar *Shigella* bivalent conjugate prepared using a different strategy designed to covalently attach OSP from *S. flexneri* 2a or *S. sonnei* to tetanus toxin through a linker exhibited strong immunogenicity in a Phase 1/2, randomized, double-blind, placebo-controlled trial, especially after two doses. It also exhibited age-dependent increases in antibody titers, showing potential as a pediatric vaccine to combat *Shigella* infections.^31^ Several other strategies, such as bioconjugation or the use of synthetic oligosaccharides, are also currently being assessed as promising techniques for developing *Shigella* conjugate vaccines.^4^^,^^32^ Although this is a promising pathway for next-generation vaccines, attaining multivalency and scale-up remain critical hurdles.

Other conjugate vaccines are highly protective in young children in resource-limited settings against invasive bacterial pathogens, where protective immunity primarily targets polysaccharide antigens. Conjugate vaccines are used globally to prevent diseases caused by pneumococci, meningococci, *Haemophilus influenzae* B, and typhoid fever in young children.^33^^–^^36^

We have previously developed and described a platform polysaccharide protein carrier conjugation system that uses squaric acid chemistry to link bacterial OSP to a carrier protein.^6^^,^^10^^–^^12^^,^^37^^–^^41^ This platform uses a single active glucosamine in the oligosaccharide core of certain Gram-negative bacteria.^6^^,^^10^^,^^11^^,^^41^ It results in single-point attachment to a carrier protein, creating a sunburst display of OSP that immunologically presents the antigen as it appears on the surface of bacteria. This approach is scalable and reproducible, and its products are easily characterized.^6^^,^^10^^,^^11^ We have previously used this approach to develop a cholera conjugate vaccine that has recently completed Phase 1 testing in humans (ClinicalTrials.gov number NCT05559983).^11^ We have similarly used this approach to develop *Shigella* conjugate vaccines that target the three most common serotypes of *S. flexneri*, which is the most common species of *Shigella* globally.^6^^,^^10^ These vaccines target *S. flexneri* serotypes 2a, 3a, and 6, and all are protectively immunogenic in mouse models.^6^^,^^10^ A vaccine that targets *S. flexneri* serotypes 2a, 3a, and 6, as well as *S. sonnei*, is predicted to protect against 80–85% of shigellosis globally.^42^^,^^43^ In our current report, we describe the evaluation of our previously described *Shigella* conjugate vaccine targeting Sf3a in a guinea pig model.^6^ This allowed us to evaluate immunogenicity in a second animal model and assess the ability of the SCV-Sf3a OSP:rTTHc vaccine to protect against mucosal–epithelial challenge in a standard animal model in the *Shigella* vaccine field: the Sereny keratoconjuctival challenge model.

We found that the SCV-Sf3a OSP:rTTHc and alum treatment was protectively immunogenic in guinea pigs. In this initial study, we evaluated the vaccine in the presence of alum adjuvant and administered alum alone as a control. In the previous analysis of cholera conjugate vaccine OSP;rTTHc, we did not observe boosting of immune responses in mice by the inclusion of alum adjuvant;^11^ however, we observed increased responses in rabbits.^37^ Based on our results in mice for SCV-Sf3a OSP:rTTHc, we anticipated the induction of prominent antigen-specific IgG responses in vaccinated guinea pigs, as well as detectable but lower-level IgM responses; this was confirmed in our guinea pig analysis. We also found that SCV-Sf3a OSP:rTTHc and alum induced OSP- and LPS-specific IgA responses. Other conjugate vaccines targeting polysaccharides of mucosal bacterial pathogens have also induced similar anti-polysaccharide IgA responses after parenteral immunization, which does not occur after parenteral vaccination with polysaccharide alone.^33^^,^^44^^,^^45^ The reason for this broadening of immune responses is not clear, but it is fortuitous because *Shigella* are mucosal pathogens. Notably, we have recently shown that *Shigella* OSP-specific IgA responses, which bind and activate polymorphonuclear cells via Fc alpha receptor (FcαR), are associated with protection against incident shigellosis in humans in Peru in a high-burden and resource-limited setting.^46^ In our current study, we did not evaluate whether the induced IgA responses were similarly functional, but we did assess bactericidal functional responses and found that SCV Sf3a OSP:rTTHc and alum induced prominent bactericidal responses in guinea pigs, which were comparable to the responses that we observed previously in our mouse studies.^6^ Such responses are largely IgG- and IgM-mediated (IgA does not bind complement in cell-free systems via the classical pathway); serum bactericidal responses have previously been found to correlate with protection against shigellosis in humans.^47^

In our current analysis, SCV Sf3a OSP:rTTHc and alum provided partial but significant protection against the most severe disease with a high dose of virulent *Shigella*. This was our first use of the model, and our challenge dose was ∼10^9^/conjunctival sac. The infectious dose 50 for *Shigella* during human intestinal infection is ∼10–100.^48^^,^^49^ Lower doses will be used in future analyses involving challenged guinea pigs, which may provide more information regarding the ability of the vaccine-induced immune response to offer protection against moderate–severe disease.

## CONCLUSION

In summary, SCV Sf3a OSP:rTTHc has been shown to be protectively immunogenic in two small animal models. Similar analyses will be performed with our other *Shigella* vaccine candidates, including monovalent and multivalent conjugate vaccines. Our results are encouraging and support the further development of the *Shigella* conjugate vaccine program.

## Supplemental Materials

10.4269/ajtmh.25-0269 Supplemental Materials
