# Supplementary material for: An O-Specific Polysaccharide Shigella flexneri 3a Conjugate Vaccine is Immunogenic and Protective against Virulent Keratoconjunctival Challenge in Guinea Pigs
Source: Am J Trop Med Hyg. 2025 Aug 5;113(4):737–43. doi: 10.4269/ajtmh.25-0269 (PMC12493252; doi:10.4269/ajtmh.25-0269)
Supplement: Supplemental Materials [file tpmd250269.SD1.pdf]

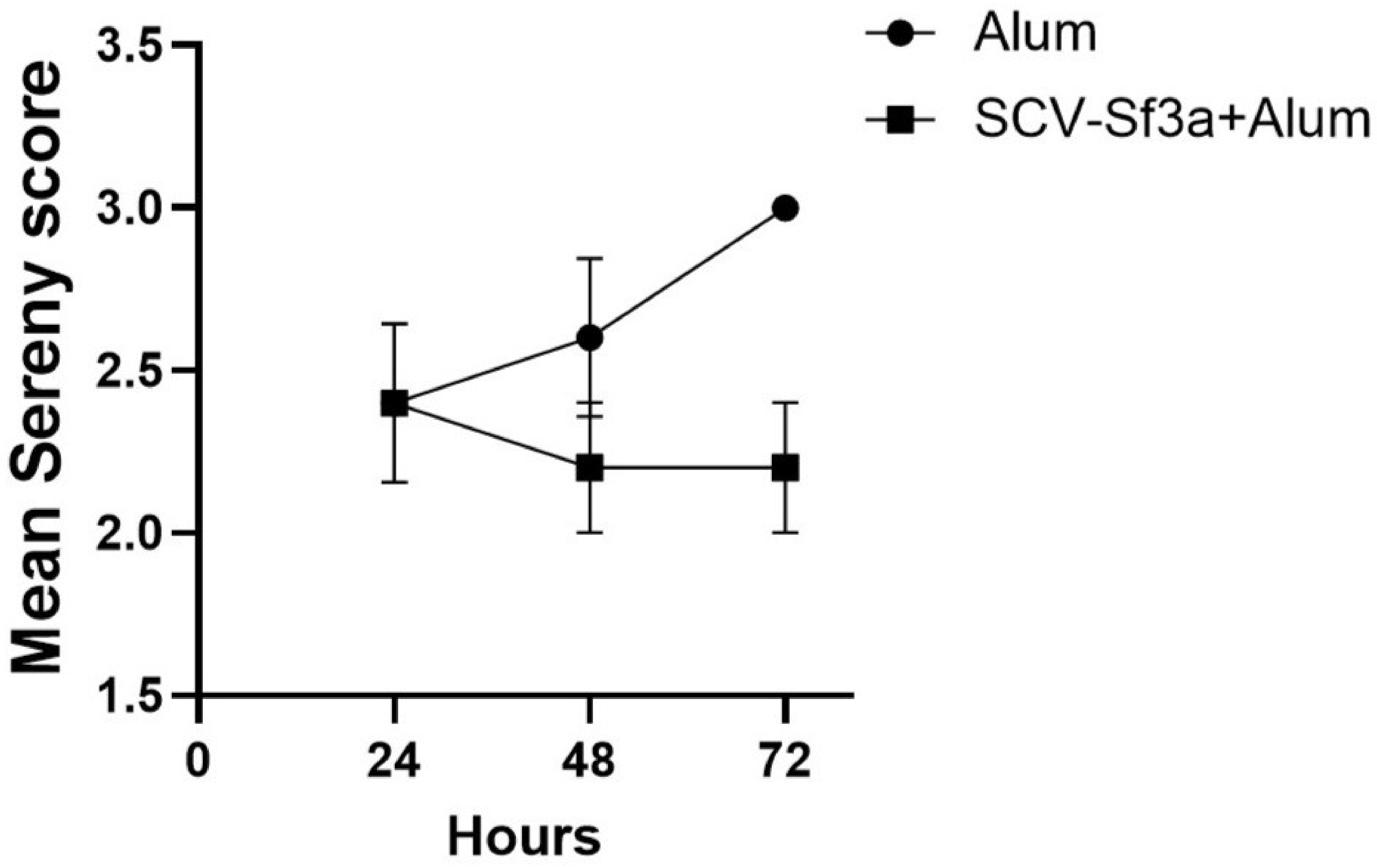

Supplemental figure S1. Mean Sereny score for cohorts based on time points. Guinea pigs (n=5 per group) were infected with $8x10^8$ cfu of *Shigella flexneri* 3a virulent strain J17B in the right eye and observed for 72 hours. [ALT-TEXT - A line graph showing the relationship between time points and mean Sereny scores of the Alum-placebo treated cohort and SCV-Sf3a vaccinated cohort.]
